# Diode Laser and Red‐Laser Photodynamic Therapy Versus Ciclopirox 8% HPCH Nail Lacquer for the Treatment of Onychomycosis: A Randomised Controlled Trial

**DOI:** 10.1111/myc.70121

**Published:** 2025-09-30

**Authors:** Sara García‐Oreja, Francisco Javier Álvaro‐Afonso, David Navarro‐Pérez, Diego León‐Herce, Aroa Tardáguila‐García, José Luis Lázaro‐Martínez

**Affiliations:** ^1^ University Podiatry Clinic, Faculty of Nursing, Physiotherapy and Podiatry Complutense University of Madrid Madrid Spain; ^2^ Diabetic Foot Unit, University Podiatry Clinic, Faculty of Nursing, Physiotherapy and Podiatry, Complutense University of Madrid Health Research Institute of the San Carlos Clinical Hospital (IdISSC) Madrid Spain

**Keywords:** ciclopirox, laser, onychomycosis, photodynamic therapy, toluidine, topical antifungal, treatment

## Abstract

**Background:**

Antifungals are the standard treatment for onychomycosis. However, oral antifungals present contraindications and potential drug–drug interactions, while topical antifungals suffer from limited efficacy and penetration. Recently, researchers have explored physical therapies, including laser and photodynamic therapy.

**Objective:**

To evaluate the clinical efficacy of combining diode laser therapy with photodynamic therapy and ciclopirox 8% hydroxypropyl chitosan (HPCH) nail lacquer in treating onychomycosis.

**Methods:**

We conducted a randomised controlled clinical trial involving patients with onychomycosis. A total of 26 patients were enrolled and followed for 12 months. Participants received either eight sessions of laser treatment combined with three sessions of photodynamic therapy, or daily treatment with ciclopirox 8% HPCH.

**Results:**

The clinical cure rate was 94.1% in the group treated with laser and photodynamic therapy, compared to 53.3% in the group treated with ciclopirox 8% HPCH (*p* = 0.008). All patients who achieved clinical cure with either treatment also reached mycologic and complete cure, with a rate of 100%. The average time to healing was significantly shorter for the group receiving laser and photodynamic therapy (3.6 ± 1.2 months) than for those treated with ciclopirox 8% HPCH nail lacquer (9.2 ± 1.6 months) (*p* < 0.001). In the laser and photodynamic therapy group, adverse events, specifically subungual hematoma and blisters, occurred in 11.4% of patients, with a recurrence rate of 33.3%. No adverse events or recurrence were observed in patients treated with ciclopirox 8% HPCH.

**Conclusions:**

Treatment of onychomycosis using diode laser and photodynamic therapy results in higher clinical cure rates and shorter healing times compared to the reference treatment with 8% ciclopirox HPCH.

**Trial Registration:**

ClinicalTrials.gov identifier: NCT05809297

## 1 Introduction

Onychomycosis is a fungal infection of the nails, predominantly affecting the toenails. It results in symptoms such as chromonychia, onycholysis, thickening and ridging of the nail plate, and subungual hyperkeratosis [1]. This condition accounts for 50% of all nail pathologies and affects approximately 5.5% of the global population [2, 3]. Additionally, the physical, aesthetic, and psychological discomfort it causes make onychomycosis a significant health concern [2, 3]. Reviews on this topic have found a psychological and psychosocial impact of up to 92%, with an impact on quality of life comparable to that of non‐melanoma skin cancer and benign tumours [2].

The primary micro‐organisms implicated in onychomycosis include dermatophytes (e.g., Trichophyton spp.), non‐dermatophyte moulds (e.g., Aspergillus spp.), and yeasts (notably, Candida spp.) [4, 5]. Before initiating treatment, confirming the diagnosis and identifying the causative fungal microorganism using microbiological tests such as culture and polymerase chain reaction (PCR) is recommended. This approach enhances cure rates and prevents resistance to antifungal drugs [6]. Additionally, numerical ratings like the Onychomycosis Severity Index (OSI) are available to assess and quantify both the severity of onychomycosis and the response to treatment [7]. The OSI is a validated scoring system, ranging from 0 to 35 points, based on specific clinical features such as affected area, proximity of the infection to the nail matrix, and the presence of dermatophytoma or hyperkeratosis larger than 2 mm. It classifies onychomycosis as mild (1–5 points), moderate (6–15 points), or severe (16 or more points) [8]. A recent study concluded that the OSI demonstrates high concordance among professionals with varying levels of clinical experience and reveals a higher prevalence of severe onychomycosis compared to clinical diagnoses, thereby guiding treatment [9].

Oral antifungal treatments are the gold standard for managing onychomycosis, particularly in severe cases, with cure rates ranging from 40% to 80%. However, these treatments can produce mild to moderate side effects, such as skin rashes, gastrointestinal disorders, and headaches, as well as serious effects, including kidney or liver damage, heart failure, and drug–drug interactions [10, 11, 12, 13]. Additionally, recent research highlights the growing issue of antifungal resistance [6]. As a result, topical antifungals in the form of nail varnish, including amorolfine 5%, efinaconazole 10%, tavaborole 5%, and ciclopirox P‐3051, have become more prevalent [12, 14, 15]. These offer fewer adverse effects and reasonable efficacy in mild to moderate cases, but require lengthy application periods exceeding 48 weeks [12, 14, 15]. A recent clinical trial evaluated the efficacy of ciclopirox 8% water‐soluble solution, the reference product in Europe, using different vehicle solutions: hydroxypropyl chitosan (HPCH) and Ciclotech (comprising sodium lauryl sulphate, hydroxypropyl‐beta‐cyclodextrin, and poloxamer‐407) [10]. This study reported a complete cure rate (clinical cure plus negative microbiological culture) after 48 weeks of treatment of 11.1% in the ciclopirox HPCH group and 10.4% in the ciclopirox + Ciclotech group [10]. The mycological cure rate (negative culture conversion) was 46% for the ciclopirox HPCH group and 47.2% for the ciclopirox + Ciclotech group [10].

The systemic toxicity associated with oral antifungals, along with the limitations of topical antifungals, has prompted research into alternative physical therapies [12, 13]. These therapies aim to enhance the efficacy of topical treatments and may be used independently or in conjunction with topical medications [12, 13, 15, 16, 17, 18].

A systematic review published in 2025 reports that photodynamic therapy (PDT) significantly reduced the severity of onychomycosis, with a reduction in the OSI ranging from 30% to 90% and a median reduction of 70% [16]. Additionally, PDT achieved mycological cure rates of up to 100% when combined with carbon dioxide laser therapy [16]. PDT utilises light in conjunction with a topical photosensitizer to generate reactive oxygen species and free radicals, which induce apoptosis and reduce fungal burden [12]. Conversely, the specific effects of the laser on fungal cells remain unclear. Laser treatment may provide aesthetic improvements in onychomycosis and potentially alter fungal cells due to its photothermolysis property, which can result in fungal cell death [12].

Laser and PDT have several advantages over oral and topical antifungal drugs, including shorter treatment durations, improved adherence, and the absence of systemic side effects and mild local effects [13, 16, 19]. High clinical and mycological cure rates have been reported in recent clinical trials and meta‐analyses when these therapies are used alone or in combination with antifungals for onychomycosis [13, 15, 16, 19]. Despite these promising results, there is currently no standard protocol detailing the number of treatment sessions or laser parameters, such as power and wavelength, necessary to achieve clinical and mycological improvement in onychomycosis. The variability in data across recent meta‐analyses [13, 16, 19] underscores the need for randomised clinical trials to validate these findings and establish a standardised treatment protocol.

The primary objective of this study was to assess the clinical efficacy of using a diode laser in combination with photodynamic therapy and ciclopirox 8% HPCH nail lacquer for treating onychomycosis.

## 2 Research Design and Methods

### 2.1 Study Design

We conducted a randomised, controlled parallel (1:1) clinical trial involving patients with onychomycosis. This study is registered on ClinicalTrials.gov.

### 2.2 Study Population

Participants qualified for the study if they were at least 18 years old and had a confirmed case of onychomycosis in at least one toenail, verified through microbiological culture and PCR testing. An experienced investigator collected nail samples with clinical signs of onychomycosis (chromonychia, onycholysis, thickening, and ridging of the nail plate, and subungual hyperkeratosis) by cutting the nails, taking scrapings or subungual hyperkeratosis, or detritus, and milling the nail plate [1, 20]. The collected material was then placed in a Petri dish, using the method detailed in our 2024 publication [20].

For patients with two or more affected nails, each nail's treatment was recorded independently.

Participants treated with oral or topical antifungals within the last month were excluded from the study. Additionally, several conditions led to exclusion, including pregnancy or breastfeeding, peripheral vascular disease, immune disorders, treatment with immunosuppressants, central or peripheral neuropathy, coagulation disorders, Raynaud's syndrome, and any alterations in the perception of cold or heat.

### 2.3 Recruitment and Randomization of Participants

Recruitment and follow‐up of all participants occurred from September 2023 to January 2025. The study received approval from a local ethics committee (Hospital Clínico San Carlos, Madrid, Spain) in February 2023 (protocol number 23/061‐EC_M) and from the Spanish Agency for Medicines and Health Products (No. EudraCT 2022‐003913‐12). Each patient provided written informed consent before inclusion in the study.

All patients in the study were monitored over a 12‐month period and assessed at 3, 6, 9, and 12 months, irrespective of their healing timeline.

Patients were randomly assigned to receive either diode laser and photodynamic therapy or ciclopirox 8% HPCH treatment. The randomization was conducted using Epidat version 4.1 (Consellería de Sanidade, Xunta de Galicia, Spain).

### 2.4 Intervention

The same podiatrist, who also served as the researcher, performed all interventions during the study.

In the study, all patients received the same hygiene recommendations, irrespective of their treatment group. These recommendations included washing the foot daily with acid pH soap (5.5), thoroughly drying the feet, disinfecting footwear daily, discarding old and contaminated footwear, not sharing footwear, minimising nail microtrauma by ensuring a proper footwear fit, and avoiding walking barefoot in public showers and swimming pools.

Group 1, which received laser and photodynamic therapy, had visits scheduled every 7 days at baseline and continued for 9 weeks. In contrast, Group 2, which was treated with ciclopirox 8% HPCH nail lacquer, had monthly visits throughout the 12‐month follow‐up.

During each visit or check‐up, debridement was performed. This procedure involved cutting and milling the detached nail plate and using a number 15 scalpel to delaminate the peri‐ and subungual hyperkeratotic tissue. These steps were undertaken to enhance the treatment effects [21]. In addition to corresponding debridement and treatment, several photographs were taken of the study nail during each visit for photographic documentation.

#### 2.4.1 Diode Laser and Photodynamic Therapy

The diode laser, operating at various wavelengths (810–1064 nm), was used in conjunction with photodynamic therapy (PDT) employing a red laser (635 nm). Both procedures utilized the Rapido Podia Diode Laser (Medency Srl, Vicenza, Italy). The DIRECTO collimated handpiece (Medency Srl, Vicenza, Italy), featuring a 1 cm^2^ spot, served as the delivery system. It was positioned approximately 1 cm from the nail plate during the laser and PDT applications.

Eight treatment sessions were conducted according to the manufacturer's recommendations and the protocol outlined in a recent case series [15].

In sessions 1, 4, and 8, we implemented the PDT‐ONYCHOMYCOSIS protocol, which combines diode laser and photodynamic therapy using variable wavelengths and durations. This protocol includes the following steps: (1) mechanical debridement of the nail, (2) disinfection with alcohol, (3) application of an 810 ± 10 nm diode laser, within 1–5 W power and a frequency range from continuous wave (CW) to 10 Hz, for 3 min to prevent thermal shock, (4) application of a topical photosensitizer, toluidine blue gel, with the nail covered for 5 min to prevent activation by natural light, (5) activation of the topical photosensitizer using a 635 ± 10 nm diode laser at 0.2 W‐CW for 10 min, (6) cleaning of the area, and (7) application of a 1064 ± 10 nm diode laser with 6–8 W power and a frequency range from CW to 10 Hz for 3 min.

The remaining five sessions involved the application of diode laser treatments using different wavelengths, following the LASER‐ONICOMICOSIS protocol. The procedure included: (1) the mechanical debridement of the nail; (2) disinfection of the nail with alcohol; (3) application of an 810 ± 10 nm diode laser with a power range of 1 W to 5 W and a frequency range from CW to 10 Hz for 3 min; (4) a 3‐min pause; and (5) application of a 1064 ± 10 nm diode laser with a power range of 6 to 8 W and a frequency range from CW to 10 Hz for 3 min.

#### 2.4.2 Ciclopirox 8% HPCH Nail Lacquer

During the initial study visit, the patient received Ony‐tec (Almirall S.A., Barcelona, Spain) for daily self‐administration at home. Ony‐tec is an antifungal nail lacquer that contains ciclopirox 80 mg/g alongside ethyl acetate, ethanol (96%), ketostearyl alcohol, hydroxypropyl chitosan, and purified water [22].

Ony‐Tec nail varnish is recommended for application in the evening, before going to bed. According to the manufacturer, the varnish requires about 30 s to dry and should not be washed off for at least 6 h [22]. Moreover, Ony‐Tec does not require removal with solvents or abrasive products, such as a nail file. Instead, it is recommended to wash the nails carefully with water before each application. Typically, complete healing of fingernails is achieved in approximately 6 months, while toenails require 9 to 12 months. Therefore, in this study, the application was maintained for a minimum of 9 months [22].

### 2.5 Outcome Measurements

#### 2.5.1 Primary Outcome

The primary outcome was the clinical cure of the affected nail following eight treatment sessions in Group 1 (diode laser and photodynamic therapy) and after at least 9 months of treatment in Group 2 (ciclopirox 8% HPCH nail lacquer).

Clinical cure is defined as the absence of the most common signs and symptoms of onychomycosis, namely subungual detritus, dermatophytoma, changes in nail plate colour, and onycholysis.

The persistence of clinical signs of onychomycosis after eight treatment sessions in Group 1 or after 12 months of treatment in Group 2 was classified as not cured.

In addition, microbiological cultures were performed on all clinically cured nails to determine the clinical and complete cure rates. A mycological cure was defined by a negative microbiological culture result. PCR was not used to assess mycological cure because it is a highly sensitive test that can detect DNA from non‐viable fungal cells, potentially leading to false‐positive results after treatment [23]. Complete cure of onychomycosis was defined as the simultaneous achievement of both clinical and mycological cure.

#### 2.5.2 Secondary Outcomes

Secondary outcomes included: (a) identifying associations between onychomycosis cure and various factors such as age, sex, history of onychomycosis, number and location of affected nails, type and severity of onychomycosis (OSI) before treatment, causal fungal organism, duration of the condition, prior treatments, type of previous treatment, and the assigned treatment; (b) time to clinical cure; (c) improvement in OSI; and (d) occurrence of recurrences after cure and during the 12‐month follow‐up.

The severity of onychomycosis using OSI was analyzed by a blinded external evaluator using photographs of the nail taken before treatment and at 3, 6, 9, and 12 months after the start of treatment.

### 2.6 Statistical Analysis

All analyses were conducted on an intention‐to‐treat basis, incorporating all patients in their assigned randomised groups. Statistical analysis was performed using SPSS v.22 (IBM Corp.). Frequency and descriptive analyses were included in the study.

The Shapiro–Wilk test was conducted to determine if the variables followed a normal distribution (*p* > 0.05), suitable for parametric tests, or a non‐normal distribution (*p* < 0.05) necessitating non‐parametric tests. The results indicated that all quantitative variables analyzed exhibited a non‐normal distribution (*p* < 0.05). Consequently, we employed non‐parametric tests for their analysis.

The primary analysis compared the clinical cure rate of onychomycosis between the two randomised groups using a chi‐square (*χ* ^2^) test. A logistic regression model was applied to analyse both qualitative and quantitative variables. For additional variable comparisons, the *χ* ^2^ test was used for qualitative variables, Spearman's test was employed for quantitative variables, and the Mann–Whitney *U* test was used when one variable was qualitative and the other quantitative. The time to cure was described using the Kaplan–Meier method, and the log‐rank test was employed to compare survival times between treatments. Statistical significance was determined at *p* < 0.05, with a 95% confidence level.

The sample size was determined to be 13 patients per treatment group. This calculation was based on achieving a statistical power of 0.80 and a significance level (alpha) of 0.05, utilising the GRANMO Sample Size Calculator version 7.12 Online (Institut Municipal d'Investigació Mèdica, Barcelona, Spain).

## 3 Results

**FIGURE 1:**
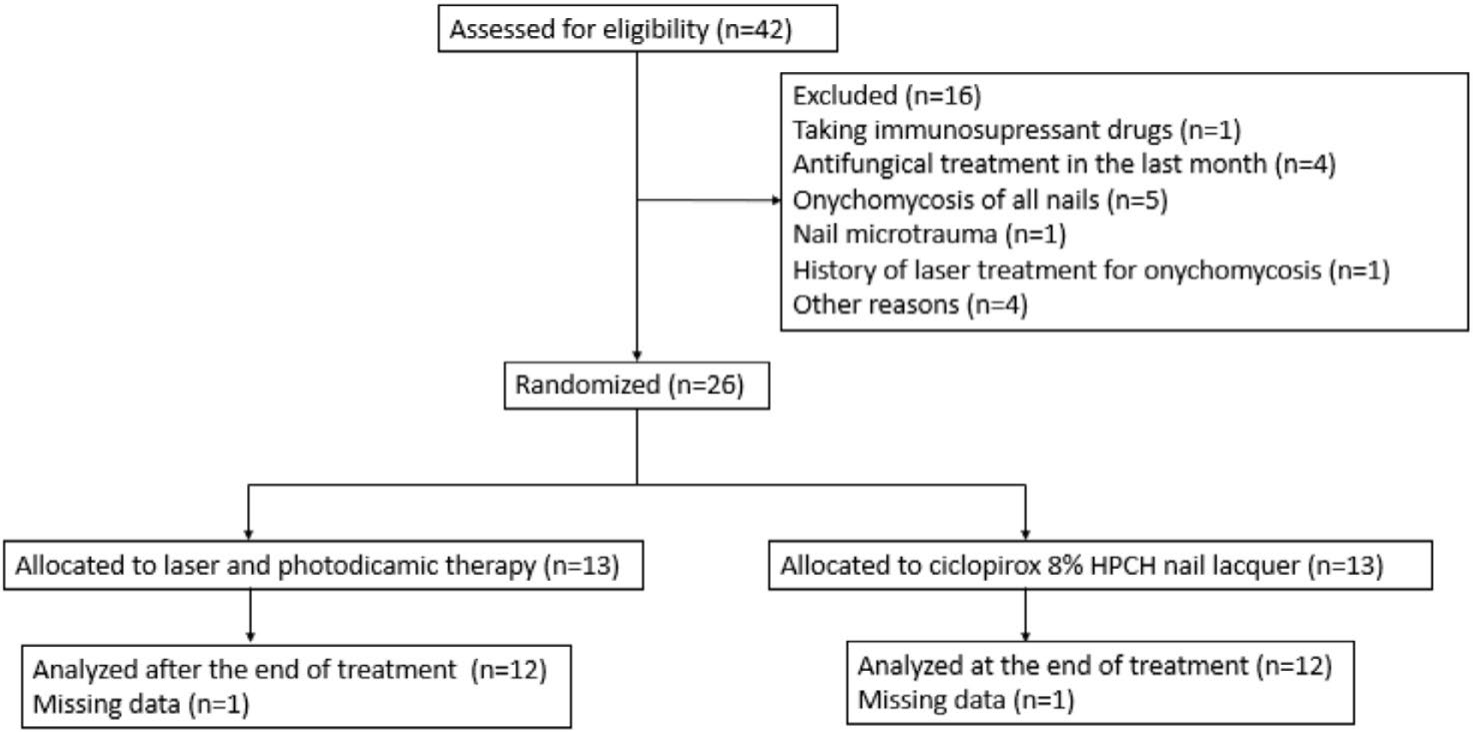
Study flow diagram.

Forty‐two patients were selected as potential participants, and 26 (61.9%) were randomly assigned to two treatment groups: 13 patients were placed in the laser and photodynamic therapy group, and 13 patients were assigned to the ciclopirox HPCH nail lacquer group. Figure 1 presents a flow chart of the study.

Thirteen patients, accounting for 18 nails (51.4%), received treatment with laser and photodynamic therapy, while another 13 patients, with 17 nails (48.6%), were treated with ciclopirox 8% HPCH nail lacquer. By the end of the study, 24 participants had completed treatment across 32 nails. Specifically, 12 patients with 17 nails finished treatment involving laser and photodynamic therapy, and another 12 patients with 15 nails concluded treatment with ciclopirox 8% HPCH.

Table 1 presents the baseline characteristics of the participants and the nails included in the study. Table 2 outlines the characteristics of their onychomycosis. Furthermore, Table 3 details the micro‐organisms identified through microbiological culture and PCR.

**TABLE 1:** Baseline characteristics of participants.

Characteristics	Diode laser and PDT (13)	Ciclopirox 8% HPCH (13)
*No. (%) of each sex*		
Female	6 (42.9)	7 (53.8)
Male	8 (57.1)	6 (46.2)
*Age (years)*		
Mean (SD)	61.9 (13.8)	61.6 (11.9)
Median (range)	67.5 (36.0–75.0)	65.0 (29.0–78.0)

**TABLE 2:** Characteristics of onychomycosis and summarises the treatment details for each group.

Characteristics	Diode laser and PTD (18)	Ciclopirox 8% HPCH (17)	*p*
*No. of nails affected per participant*			
Mean (SD)	1.4 (0.5)	1.8 (1.6)	0.739
Median (range)	1.0 (1.0–2.0)	1.0 (1.0–7.0)	
*Number of participants with 2 or > 2 affected nails*	6 (42.9)	6 (46.2)	0.863
*No. (%) of participants with a history of onychomycosis*	5 (35.7)	3 (23.1)	0.472
*Duration of onychomycosis*	No. of nails (%)	No. of nails (%)	
0–1 months	0 (0.0)	0 (0.0)	0.341
2–3 months	1 (5.6)	2 (11.8)	
4–6 months	1 (5.6)	1 (5.9)	
7–12 months	0 (0.0)	0 (0.0)	
12–24 months	3 (16.7)	0 (0.0)	
> 24 months	13 (72.2)	14 (82.4)	
*No. (%) of nails previously treated*	16 (88.9)	11 (64.7)	0.089
*Type of previous treatment*	No. of nails (%)	No. of nails (%)	
Oral antifungal	3 (17.6)	0 (0.0)	**0.025**
Topical antifungal	10 (58.8)	8 (72.7)	
Combined oral and topical antifungal treatment	4 (23.5)	0 (0.0)	
Laser	0 (0.0)	0 (0.0)	
Chemical avulsion	0 (0.0)	0 (0.0)	
Tea tree oil	0 (0.0)	3 (27.3)	
*Location*	No. of nails (%)	No. of nails (%)	
Hallux right foot	6 (33.3)	5 (29.4)	0.529
Lesser toes right foot	3 (16.8)	0 (0.0)	
Hallux left foot	9 (50.0)	10 (58.8)	
Lesser toes left foot	0 (0.0)	2 (11.8)	
*Clinical signs*	No. of nails (%)	No. of nails (%)	
Onycholysis	15 (83.3)	17 (100.0)	0.078
Onychogryphosis	13 (72.2)	9 (52.9)	0.238
Subungual hyperkeratosis	12 (66.7)	14 (82.4)	0.289
Periungual hyperkeratosis	6 (33.3)	7 (41.2)	0.631
Chromonychia	18 (100.0)	17 (100.0)	—
Dermatophytoma	8 (44.4)	2 (11.8)	**0.032**
*Type of onychomycosis*	No. of nails (%)	No. of nails (%)	
Distal	7 (38.9)	6 (35.3)	0.879
Distal‐lateral	4 (22.2)	3 (17.6)	
Total dystrophic	7 (38.9)	8 (47.1)	
*OSI*	No. of nails (%)	No. of nails (%)	
Mild	3 (16.7)	0 (0.0)	0.209
Moderate	4 (22.2)	5 (29.4)	
Severe	11 (47.8)	12 (70.6)	
*Time to clinical cure (months)*			
Mean (SD)	3.6 (1.8)	4.8 (1.8)	**< 0.01**
Median (range)	4.0 (0.0–6.0)	5.0 (1.0–6.0)	

*Note:* Bold value statistically significant *p* < 0.05.

**TABLE 3:** Summarises of fungal type and micro‐organisms isolated by culture + PCR.

Type of fungus	Microorganism	Total *N* (%)	Diode laser and PDT (18)	Ciclopirox 8% HPCH (17)
			*N* (%)	*N* (%)
Dermatophyte		28 (80.0)	16 (88.9)	12 (70.6)
	Trichophyton rubrum	5 (14.3)	4 (22.2)	1 (5.9)
	Trichophyton tonsurans	2 (5.7)	0 (0.0)	2 (11.8)
	Trichophyton mentagrophytes	5 (5.7)	1 (5.6)	1 (5.9)
	Not isolated by culture, detected through PCR	19 (52.3)	11 (61.1)	8 (47.1)
Yeast		3 (8.6)	0 (0.0)	3 (17.6)
	Candida sp.	3 (8.6)		3 (17.6)
Mould		4 (11.4)	2 (11.8)	2 (11.8)
	Penicilum	2 (5.7)	0 (0.0)	2 (11.8)
	Cladosporium spp.	2 (5.7)	2 (11.1)	0 (0.0)

Of the 32 treated nails, 24 (68.6%) achieved a clinical cure after treatment. Specifically, 16 out of 32 (67.7%) nails belonged to the laser and photodynamic therapy group, while 8 (33.3%) were from the ciclopirox 8% HPCH nail lacquer group. The clinical cure rate was 94.1% (16 out of 17 nails) in the laser and photodynamic therapy group compared to 53.3% (eight out of 15 nails) in the ciclopirox 8% HPCH nail lacquer group, showing significant differences between the groups (difference of 40.8%, *p* = 0.008). Logistic regression analysis further supported the difference between these treatment groups (OR: 0.071 [95% confidence interval: 0.007–0.685], *p* = 0.022). Microbiological cultures conducted after clinical cure confirmed negative results in all 24 nails tested, thus indicating a 100% rate of both mycological and complete cure in these patients.

Table 4 presents the results of the univariate analysis, which identified a statistical association between having a history of onychomycosis and treatment outcomes. Specifically, a higher percentage of patients who were not cured had previously experienced the disease.

**TABLE 4:** Results univariate model.

Variables	Cured (24)	Uncured (8)	All participants		
	Frequency (%)	Frequency (%)	OR	95% CI	*p*
*Sex*					
Female	13 (81.3)	3 (18.8)	0.508	0.09–2.62	0.418
Male	11 (68.8)	5 (31.3)			
*Two or > 2 affected nails*	11 (61.1)	7 (38.9)	8.273	0.88–78.01	0.065
*Previous onychomycosis*	4 (40.0)	6 (60.0)	15.0	2.18–103.04	**0.006**
*Time of evolution*					
0–1 months	0 (0.0)	0 (0.0)	—	—	0.998
2–3 months	2 (66.7)	1 (33.3)			
4–6 months	1 (100.0)	0 (0.0)			
7–12 months	0 (0.0)	0 (0.0)			
12–24 months	3 (100.0)	0 (0.0)			
> 24 months	18 (72.0)	7 (28.0)			
*Previous treatment*	17 (70.8)	7 (29.2)	2.882	0.29–27.97	0.361
*Type of previous treatment*					
Oral antifungal	2 (66.7)	1 (33.3)	—	—	1.0
Topical antifungal	12 (66.7)	6 (33.3)			
Combined oral and topical antifungal treatment	3 (100.0)	0 (0.0)			
Laser	0 (0.0)	0 (0.0)			
Chemical avulsion	0 (0.0)	0 (0.0)			
Tea tree oil	1 (100.0)	0 (0.0)			
*Location*					
Hallux right foot	9 (90.0)	1 (10.0)	—	—	0.974
Lesser toes right foot	2 (66.7)	1 (33.3)			
Hallux left foot	12 (70.6)	5 (29.4)			
Lesser toes left foot	1 (50.0)	1 (50.0)			
*Clinical signs*					
Onycholysis	21 (72.4)	8 (27.6)	—	—	0.999
Onychogryphosis	15 (71.4)	6 (28.6)			0.522
Subungual hyperkeratosis	16 (66.7)	8 (33.3)			0.999
Periungual hyperkeratosis	8 (61.5)	5 (38.5)			0.156
Chromonychia	24 (75.9)	8 (25.0)			—
Dermatophytoma	8 (88.9)	1 (11.1)			0.277
*Type of onychomycosis*					
Distal	10 (76.9)	3 (23.1)	—	—	0.701
Distal‐lateral	6 (100.0)	0 (0.0)			
Total dystrophic	8 (61.5)	5 (38.5)			
*Fungal type*					
Dermatophyte	21 (84.0)	4 (16.0)	—	—	0.113
Yeats	1 (33.3)	2 (66.7)			
Mould	2 (50.0)	2 (50.0)			
*OSI*					
Mild	3 (100.0)	0 (0.0)	—	—	0.452
Moderate	8 (88.9)	1 (11.1)			
Severe	13 (65.0)	7 (35.0)			
*Adverse effects*	4 (100.0)	0 (0.0)	—	—	0.999

	Mean (SD)	Mean (SD)	OR	95% CI	*p*
Age	64.1 (12.1)	61.5 (11.2)	1.018	0.95–1.09	0.588
No. of nails	1.5 (0.5)	3.1 (2.4)	0.169	0.02–1.63	0.124
OSI	15.7 (9.0)	20.4 (6.6)	0.934	0.84–1.03	0.187

*Note*: Bold value indicates statistically significant *p* < 0.05.

Abbreviations: CI, confidence interval; OR, odds ratio.

**FIGURE 2:**
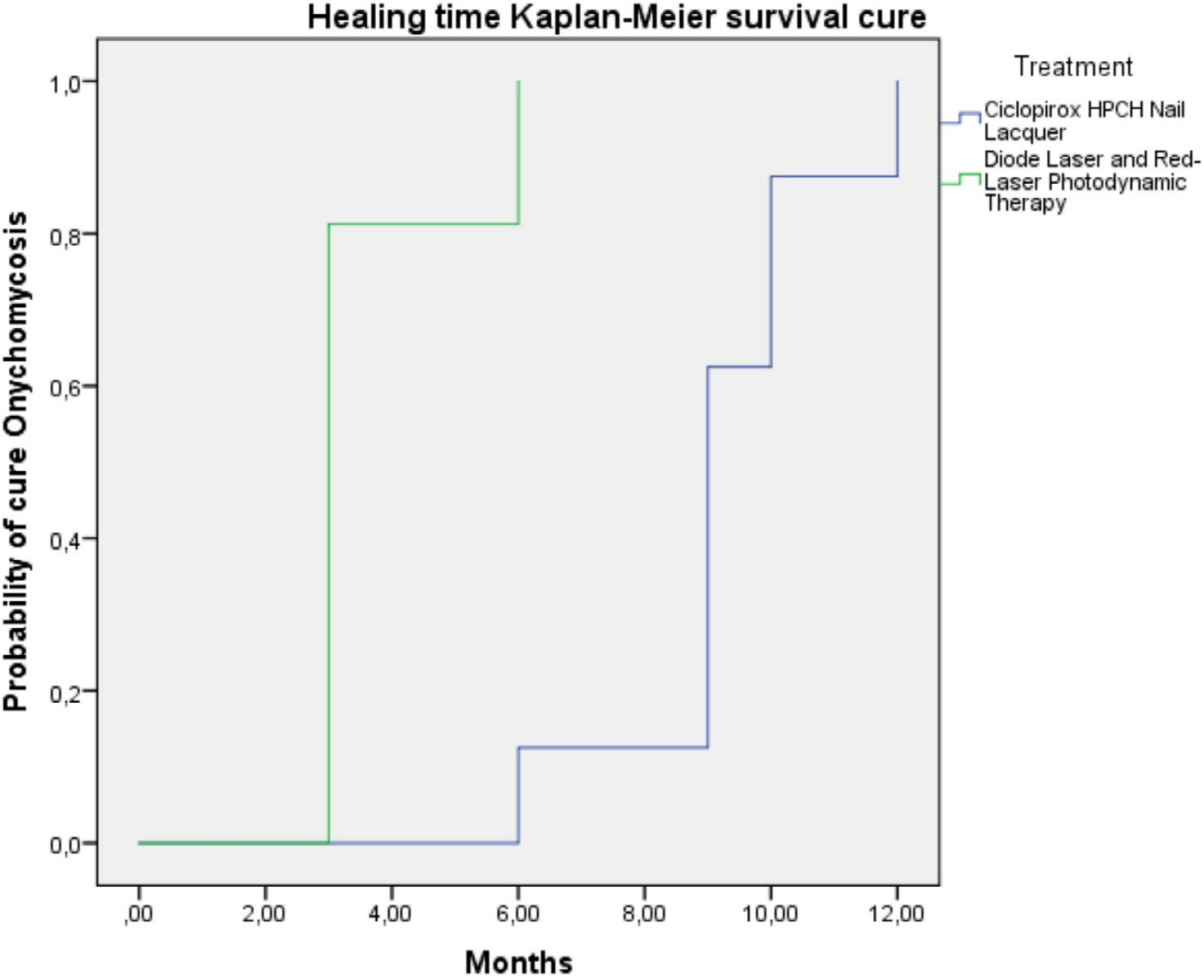
Kaplan–Meier survival curve. Healing time (months) of onychomycosis by treatment group.

Table 2 summarises the treatment details for each group. Differences among the groups were noted in the presence of dermatophytoma and the type of previous treatment received. Additionally, the mean time to healing was shorter for the group receiving laser treatment and photodynamic therapy (3.6 ± 1.2 months) compared to the group treated with ciclopirox % HPCH nail lacquer (9.2 ± 1.6 months) (*p* < 0.001). Figure 2 illustrates significant differences in the probability of cure among the treatment groups, as determined using the Kaplan–Meier method for time to cure and the log‐rank test (*p* < 0.001).

Table 5 presents the mean OSI scores before the initiation of each treatment and at 3, 6, 9, and 12 months into each treatment. Comparing the mean scores at baseline with those at 3, 6, 9, and 12 months post‐treatment revealed no statistically significant differences between the two groups (*p* > 0.05). However, a significant improvement in OSI scores was observed across all patients at 3 months (*p* = 0.01). Specifically, significant improvements were noted in both Group 1 (*p* < 0.01) and Group 2 (*p* = 0.032) at 3 months and in Group 1 at 6 months (*p* = 0.02) compared to baseline scores.

**TABLE 5:** OSI scores before baseline and after each treatment.

		Total	Diode laser and PDT	Ciclopirox 8% HCPH
OSI pre‐treatment	Mean (SD)	17.1 (8.3)	16.6 (9.8)	17.7 (6.6)
	Median (range)	16.0 (1.0–35.0)	16.0 (1.0–35.0)	18.0 (6.0–30.0)
OSI 3 months	Mean (SD)	5.9 (5.0)	6.0 (5.4)	5.9 (4.8)
	Median (range)	4.0 (1.0–20.0)	3.5 (1.0–20.0)	4.0 (1.0–16.0)
OSI 6 months	Mean (SD)	5.7 (6.3)	5.0 (4.6)	6.6 (7.6)
	Median (range)	4.0 (0.0–30.0)	4.0 (0.0–18.0)	4.0 (1.0–30.0)
OSI 9 months	Mean (SD)	5.3 (5.8)	7.3 (0.0–18.0)	5.3 (5.2)
	Median (range)	2.0 (0.0–18.0)	3.0 (0.0–18.0)	4.0 (0.0–16.0)
OSI 12 months	Mean (SD)	1.8 (4.1)	6.4 (7.8)	4.0 (5.8)
	Median (range)	0.0 (0.0–16.0)	1.0 (0.0–18.0)	1.0 (0.0–16.0)

Adverse effects were observed in four nails out of 35, involving two patients out of 26 in the study. These effects were mild, consisting of subungual hematoma and subungual blisters, and occurred in patients who received laser and photodynamic therapy (*p* = 0.039). These adverse effects appeared in 11.4% of the treated nails, although they did not seem to affect healing outcomes (*p* > 0.05). Recurrence of onychomycosis occurred in six of the 24 cured nails during the 12‐month follow‐up, all of which had received laser and photodynamic therapy (*p* = 0.46). Among the 12 patients who completed treatment with this method, four experienced recurrences, resulting in a 33.3% recurrence rate. No association was found between the occurrence of adverse effects and subsequent relapses (*p* > 0.05).

## 4 Discussion

This study aimed to compare the clinical efficacy of diode laser combined with photodynamic therapy to ciclopirox 8% HPCH nail polish in treating onychomycosis. Significant differences were found between the two treatments, with a difference of 40.8% (*p* = 0.008). The clinical cure rate was higher in the group treated with laser and photodynamic therapy, achieving 94.1%, compared to 53.3% in the group treated with ciclopirox 8% HPCH nail varnish. All patients who achieved clinical cure also exhibited mycological and complete cure, irrespective of the treatment method. No significant differences were observed between the two treatment groups in OSI scores at 3, 6, 9, and 12 months. All patients demonstrated improved mean OSI scores at 3 and 6 months of treatment, regardless of the treatment, and only patients who received laser‐PDT treatment had a significant improvement in OSI at 6 months (Table 5).

To our knowledge, no previous studies have compared laser treatment directly with the topical application of ciclopirox 8%, the reference product in Europe. However, clinical trials and meta‐analyses have compared the efficacy of onychomycosis treatments involving different types of lasers and medical interventions, such as oral antifungals and other topical agents like amorolfine.

The systematic review and meta‐analysis by Meretsky et al. [13] found that various laser treatments resulted in significantly higher clinical and mycological cure rates than oral antifungals, such as terbinafine and itraconazole, and topical treatments like amorolfine over follow‐up periods of 12 to 24 weeks. This analysis reported high heterogeneity among the studies included (*I* ^2^ = 68%) [13].

Another meta‐analysis on laser therapy conducted by Yeung et al. [18] examined clinical, mycological, and complete cure rates across 22 prospective studies, including four randomised controlled trials (RCTs) and 18 uncontrolled trials. When analysing patients as the unit of analysis, the clinical cure rate was 67.2%, the mycological cure rate was 70.4%, and the complete cure rate was 7.2%, with substantial heterogeneity (clinical improvement: *I* ^2^ = 69%; mycological cure: *I* ^2^ = 88%; complete cure: *I* ^2^ = 60%) [18]. Conversely, when nails were considered the unit of analysis, the clinical cure rate was 56.2%, the mycological improvement rate was 22.9%, and the complete cure rate was 24.5% [18].

In all trials included in the Yeung et al. [18] meta‐analysis, two to eight sessions were conducted using 1064 nm Nd:YAG lasers, except for two studies that employed 870 and 930 nm infrared light and 1340 nm Nd:YAG lasers. The fluences ranged from 5 to 424 J/cm^2^, pulse durations from 0.3 to 40 ms, and temperatures from 39°C to 60°C [18].

In 2025, Navarro Pérez et al. [17] published a series of ten clinical cases involving a combined treatment for onychomycosis. This treatment included three sessions of photodynamic therapy (PDT) with toluidine blue gel and a 10‐min, 635 nm diode laser, alongside a daily home application of ciclopirox 8% HPCH for 6 months. Monthly revisions were conducted for nail plate cutting and milling over the same period. At the end of 6 months [17], all patients had negative microbiological culture results. Clinically, 90% (nine out of ten) of the patients were completely cured [17]. The mean OSI value initially was 18.50 ± 8.947, which decreased to 10.30 ± 6.129 at 3 months and further declined to 4.10 ± 4.08 at 6 months [17].

A systematic review of the efficacy of photodynamic therapy for onychomycosis reported clinical cure rates ranging from 20% to 80% and improvements in OSI values from 30% to 90% [16]. Notably, the review identified mycological cure rates of up to 100% when PDT was combined with CO_2_ fractional laser therapy [16]. The review by Alves et al. [16] included 18 studies, with PDT applied using diode lasers (450–700 nm) in 89% of the studies and intense pulsed light in the remainder. Photosensitizers used included methylene blue, aminolevulinic acid, methyl‐5‐aminolevulinate, and toluidine blue gel, among others [16]. The authors evaluated the methodological quality of these studies, rating the clinical trials as having ‘moderate’ certainty due to methodological heterogeneity and the non‐randomised studies as having ‘low’ certainty [16].

The authors of this article argue that significant heterogeneity exists among studies previously described in the literature. This variability is evident in aspects such as laser types, laser parameters (including power and wavelength), control groups, and the variables analysed. In a review by Gupta et al. [24], which evaluated the efficacy of various laser types (diode, Nd:YAG, fractionated CO_2_, and Er:YAG) across 53 studies, including 18 randomised controlled clinical trials, several conclusions were drawn. They found that the Nd:YAG and CO_2_ lasers are the most extensively studied [24]. However, they also noted that the quality of clinical trial data is generally poor, as these studies often fail to assess mycological cure [24]. Additionally, Gupta et al. pointed out that laser parameters, such as power, wavelength, application time, and the number of sessions, varied greatly across the studies [24].

Cure rates for the group treated with ciclopirox 8% HPCH nail polish were lower; however, the clinical cure rates achieved (53.3%) exceeded those reported in prior studies at 12 months of treatment. This improvement might be attributed to the combined regimen of monthly clinical debridement and recommended hygiene measures for all patients. Zalacaín et al. [10], in a clinical trial conducted on a sample of 381 patients, analyzed the efficacy of ciclopirox 8% with Ciclotech technology versus ciclopirox 8% HPCH through the complete cure rate, mycological cure rate, and percentage of clinical improvement (mycological cure and reduction of clinical signs of onychomycosis ≥ 20%) without observing significant differences between the two treatments in the complete cure rates at 52 weeks of treatment, which were 10.48% with the Ciclotech vehicle and 11.1% with HPCH. They discovered no significant differences between the two groups in either cure rates (32% and 27%) or clinical improvement percentages (27.2% and 20.6%) [10]. Similarly, Piraccini et al. [25] conducted a clinical trial in 2018 with 467 patients, reporting a complete cure rate (100% clear nail and negative culture) of 7.6% after 48 weeks of ciclopirox P‐3051 treatment, which increased to 15.1% in a follow‐up evaluation 12 weeks later (60 weeks from the start of treatment). Their findings also indicated a clinical improvement rate (clear nail > 90% and negative culture) of 31.9% at 48 weeks and 34.5% at 60 weeks, with a mycological cure rate of 91.6% at 48 weeks and 82.4% at 60 weeks [25]. Despite these findings, the 2020 Cochrane review concluded that efinaconazole, not marketed in Europe, is the most effective topical antifungal nail lacquer based on high‐level evidence. It is followed by ciclopirox P‐3051 (8% hydrolacquer), supported by moderate‐level evidence [14].

Logistic regression analysis indicated significant differences between treatment groups, with an odds ratio of 0.071 (95% confidence interval: 0.007–0.685, *p* = 0.022). Additionally, the model revealed a statistical association between cure rates and a personal history of onychomycosis. Patients with a history of the disease were less likely to be cured (*p* = 0.006). However, no significant associations were found between cure and other variables, including age, location, microorganism, clinical signs, number of affected nails, severity of onychomycosis, previous treatment received, and type of prior treatment.

The mean number of months to healing was significantly lower in the group receiving laser treatment and photodynamic therapy (3.6 ± 1.2 months) compared to the group treated with ciclopirox 8% HPCH nail lacquer (9.2 ± 1.6 months) (*p* < 0.001). The log‐rank test also indicated a higher probability of cure in the laser and photodynamic therapy group (*p* < 0.001). To the authors' knowledge, no other studies have reported the mean treatment duration in months until cure when using laser, photodynamic therapy, or ciclopirox 8%. In this study, all patients were monitored for a full 12 months, irrespective of when a cure was achieved. Most previous studies on laser treatment have involved follow‐ups of up to 24 weeks, except Zalacaín et al. [10, 13, 14, 25, 26, 27], who extended follow‐up to 18 months. For ciclopirox 8% HPCH, the follow‐up period is typically longer, ranging from 48 to 60 weeks.

During the treatment, no adverse effects were observed in the group treated with ciclopirox 8% HPCH. However, two patients in the group subjected to laser and photodynamic therapy experienced mild adverse effects, specifically subungual hematoma and blister formation. These effects were not linked to treatment outcomes or subsequent relapses (*p* > 0.05). Foley et al. [14], in their 2020 Cochrane review, did not report adverse effects associated with laser treatment for onychomycosis. They did, however, identify erythema, skin rash, and burning sensation as primary adverse effects of 8% ciclopirox hydrolacquer [14]. In contrast, Meretsky et al.'s meta‐analysis [13] reported pain as an adverse effect of laser treatment in three out of eight included studies.

Recurrence occurred in four out of 12 patients (33.3%) who completed treatment with laser and photodynamic therapy. In contrast, although the group treated with ciclopirox 8% had a lower clinical cure rate, no recurrences were observed. The authors suggest this could be attributed to the shorter follow‐up period after treatment completion in the ciclopirox 8% group, where the treatment lasted a minimum of 9 months. In a clinical trial examining the effects of varying session numbers using a 1064 nm Nd:YAG laser at 240–348 J/cm^2^, recurrence rates at week 24 were 7.1% for the group with four sessions, 9.1% for the group with eight sessions, and 1.8% for the group with 12 sessions [27].

Other findings from the statistical analysis were the higher prevalence of dermatophytoma in the group of patients treated with laser and PDT, which did not appear to influence the healing results. Previous studies have identified dermatophytomas as a sign of severe infection, showing low antifungal healing rates and being excluded from most clinical trials [21].

This study had several limitations. First, the follow‐up of all patients and the treatment for the group receiving laser and photodynamic therapy were conducted exclusively by the same podiatrist researcher at a single podiatry center specializing in onychomycosis. Thus, treatment results might vary if applied in other medical centers by different professionals. Furthermore, there was no blinding of the investigators or the patients, which could potentially introduce bias in the results of the different treatments. Second, the study did not include a third group that combined the reference treatment with the new laser plus photodynamic therapy protocol. As a result, it remains unclear whether combining these treatments could further enhance clinical, mycological, and aesthetic outcomes. Third, microbiological cultures were not conducted for patients who did not achieve clinical cure, which may introduce bias in the mycological cure rate. Finally, the post‐treatment follow‐up period was longer in the group treated with laser and photodynamic therapy because treatment in the 8% ciclopirox HPCH group was 9 months.

Despite its limitations, this study represents the first randomised controlled clinical trial comparing the efficacy of onychomycosis treatment using a combination of diode laser and photodynamic therapy with the standard treatment of 8% ciclopirox hydrolacquer. Notable strengths of the study include a 12‐month follow‐up period for all patients and the detailed description of a clinical protocol for the application of laser and/or photodynamic therapy in treating onychomycosis.

In conclusion, treating onychomycosis with a diode laser combined with photodynamic therapy over an 8‐session protocol is effective, resulting in significantly higher clinical cure rates than the reference treatment using 8% ciclopirox HPCH. Furthermore, the healing times with diode laser and photodynamic therapy are considerably shorter compared to the daily topical application of 8% ciclopirox HPCH. However, all recurrences occurred in the group treated with laser and PDT, so further research is considered necessary.

Future studies could explore the combination of laser and/or photodynamic therapy with topical antifungal treatment using 8% ciclopirox hydrolacquer. This research could analyze mycological cure rates across all cases and compare varying numbers of treatment sessions. Additionally, it would be beneficial to conduct multicenter studies with longer follow‐up periods and larger sample sizes.

## Author Contributions

**Sara García‐Oreja:** conceptualization, investigation, writing – original draft, methodology, funding acquisition, validation, visualization, writing – review and editing, software, formal analysis, project administration, data curation. **Francisco Javier Álvaro‐Afonso:** conceptualization, supervision, resources, validation, visualization. **David Navarro‐Pérez:** formal analysis, validation, visualization. **Diego León‐Herce:** data curation, investigation, validation, visualization. **Aroa Tardáguila‐García:** formal analysis, validation, visualization. **José Luis Lázaro‐Martínez:** supervision, conceptualization, funding acquisition, visualization, validation.

## Ethics Statement

This Randomised Controlled Clinical Trial was conducted at the University Podiatry Clinic of the Complutense University of Madrid, whose protocol was approved by the medical ethics committee of the Health Research Institute of the Hospital Clínico San Carlos (IdISSC) (protocol number 23/061‐EC_M) and the Spanish Agency for Medicines and Health Products (No. EudraCT 2022‐003913‐12).

## Consent

All patients voluntarily signed the consent statement for the use of their image and publication of their case details.

## Conflicts of Interest

The authors declare no conflicts of interest.

## Data Availability

The data that support the findings of this study are available from the corresponding author upon reasonable request.
